# Proton pump inhibitors as anti vacuolar-ATPases drugs: a novel anticancer strategy

**DOI:** 10.1186/1756-9966-29-44

**Published:** 2010-05-08

**Authors:** Enrico P Spugnini, Gennaro Citro, Stefano Fais

**Affiliations:** 1 S.A.F.U. Department, Regina Elena Cancer Institute, Rome, Italy; 2 Department of Therapeutic Research and Medicines Evaluation, Unit of Antitumor Drugs, Istituto Superiore di Sanità, Rome, Italy

## Abstract

The vacuolar ATPases are ATP-dependent proton pumps whose functions include the acidification of intracellular compartments and the extrusion of protons through the cell cytoplasmic membrane. These pumps play a pivotal role in the regulation of cell pH in normal cells and, to a much greater extent, in tumor cells. In fact, the glucose metabolism in hypoxic conditions by the neoplasms leads to an intercellular pH drift towards acidity. The acid microenvironment is modulated through the over-expression of H^+ ^transporters that are also involved in tumor progression, invasiveness, distant spread and chemoresistance. Several strategies to block/downmodulate the efficiency of these transporters are currently being investigated. Among them, proton pump inhibitors have shown to successfully block the H^+ ^transporters in vitro and in vivo, leading to apoptotic death. Furthermore, their action seems to synergize with conventional chemotherapy protocols, leading to chemosensitization and reversal of chemoresistance. Aim of this article is to critically revise the current knowledge of this cellular machinery and to summarize the therapeutic strategies developed to counter this mechanism.

## Review

Tumor cells rely on H^+ ^exchangers to relieve themselves from the dangerous protons byproduct of cancer metabolism that could trigger a cascade of lytic enzymes that ultimately would lead to self-digestion. Among these the most investigated are the vacuolar H^+^-ATPases (V-ATPases). V-ATPases are ATP dependent H^+ ^transporters that utilize the energy freed by the hydrolysis of ATP with the active transport of protons from the cytoplasm to the lumen of intracellular compartments or, if located within the cytoplasmic membrane, the extracellular compartment [1-4]. Structurally speaking, the V-ATPases are composed of a peripheral domain (V_1_) that carries out ATP hydrolysis and an integral domain (V_0_) responsible for exchanging protons. The peripheral domain is made up of eight subunits (A-H) while the integral domain contains six subunits (a, c, c', c", d and e). V-ATPases work through a rotary mechanism in which ATP hydrolysis within V_1 _promotes the rotation of a central rotary domain, relative to the remainder of the complex, while the rotation of a proteolipid ring belonging to V_0 _domain moves protons through the membrane [5-7]. Two important physiological mechanisms of regulating V-ATPase activity *in vivo *are reversible dissociation of the V_1 _and V_0 _domains and changes in coupling efficiency of proton transport and ATP hydrolysis [8-15]. Malignant tumor cells overexpress lysosomal proteins on the cell surface, with deranged lysosomal activities, including acidification of internal vesicles, possibly involving altered V-ATPase function [16,17]. The acidic tumor environment is a consequence of anaerobic glucose metabolism with secondary production of lactates byproducts through the upregulation of hypoxia-inducible factor 1α [18] or can be due to inadequate tumor perfusion, hypoxia secondary to disordered tumor growth or enhanced transmembrane pH regulation[19]. These pumps, coupled with other ion exchangers, play a key role in the establishment and maintenance of malignant tumor environment and promote the selection of more aggressive cell phenotypes able to survive in this highly selective ambient.

## Role of V-ATPases in tumor spread

V-ATPases play a critical role in the maintenance of an appropriate relatively neutral intracellular pH, an acidic luminal pH, and an acidic extracellular pH by actively pumping protons either through ion exchange mechanisms or by segregating H^+ ^within cytoplasmic organelles that are subsequently expelled [20]. It is hypothesized that the low extracellular pH of tumors might trigger proteases, leading to the dissolution of extracellular matrix. This phenomenon, as is well known, significantly contributes to tumor invasion and dissemination [21,22]. In fact, tumor invasion depends on tumor acidifying ability that ultimately leads to secretion and activation of several classes of proteases [23,24]. It is indeed known that low extracellular pH can trigger several proteases such as MMP-2, MMP-9, cathepsin B, and cathepsin L and result in acidity-induced up-regulation of the proangiogenic factors VEGF-A and IL-8 [25,26]. As a consequence, the neutralization of these mechanisms has been actively pursued by many investigators who have been only partially successful, since so far it has been possible to block one or more MMPases but not all them simultaneously [27]. A recent publication points out that by inhibiting of V-ATPases through RNA interference, it was possible to prevent cancer metastases in a murine model [28]. This approach offers a new strategy to cope with the process of tumor spread (that is mediated by a continuous process of extracellular matrix degradation and tumor angiogenesis) by raising the extracellular tumor pH, thus arresting the activation of matrix degradating proteases. Finally, besides being a potential target of anticancer drugs, it is conceivable that V-ATPases might become a predictive factor of tumor behaviour and final outcome through the immunohistochemical evaluation of their expression and cellular distribution in tumor biopsies [29-31].

## Role of V-ATPases in chemoresistance

The acidic microenvironment caused by changes in the pH gradient between the intracellular and the extracellular compartments as well as the pH gradient between the cytoplasm and the intracellular organelles can be significantly involved in the mechanism of drug resistance [32,33]. There are several mechanisms involved in this phenomenon, including decreased uptake or neutralization of weakly basic drugs by the acidic tumor microenvironment or the sequestration of chemotherapy drugs within lysosomal vesicles [32-36]. An accelerated turnover of acidic vesicles may represent an additional tumor strategy of drug resistance based on counteracting current transportation [37]. Several investigators developed new approaches to better characterize tumor pH in animal models [38,39] mostly through imaging systems in order to identify novel targets. As a result, new approaches have been developed to modulate drug efficacy within the low pH tumor milieu including the use of RNA interference, bicarbonates or the induction of metabolic alkalosis [40-43]. Finally, two recently published articles describe the chemosensitizing action of proton pump inhibitors (omeprazole) in a murine model of orthotopic cutaneous melanoma, a well known chemo-refractory neoplasm, opening a novel field of investigation [44,45].

## Pump inhibitors as antitumor drugs

The various functions played by V-ATPases in tumors, including proliferation, tumorigenesis, drug resistance and tumor progression, make them potential targets for preclinical investigators and clinicians. The evidence that the expression of such proteins within tumor cells is increased in chemoresistant phenotypes and the fact that this expression could be induced by anticancer drugs, prompted oncologists to pursue the pharmaceutical neutralization of this tumor function [46-48]. Molecular and pharmacological therapy of these biological targets is technically extremely difficult and may carry a significant degree of toxicity. On the other hand, proton pump inhibitors are normally adopted in the treatment of gastritis, Zollinger-Ellison syndrome and, limitedly to veterinary oncology, gastric hyperacidity secondary to mast cell tumors in dogs and cats [49]. These drugs have been shown to be highly effective at inhibiting V-ATPases in vitro and well tolerated and extremely efficacious in murine models, resulting in increased chemotherapy efficacy and improved tumor control [44,45,50]. Moreover, the same schedule has been able to revert chemoresistance to 5 fluorouracil, cisplatin and doxorubicin resulting in a caspase-independent cell death. Table 1 summarizes the different efflux pumps identified so far within tumor cells and their role in the maintenance of acid-base homeostasis and provides a short list of references for each pump [21,35,51-59].

**Table 1:** Efflux pumps described as hyperexpressed and/or hyperfunctional in malignant tumor cells or tumors

Type of pump	Cellular localization	Function	References
H+ATPase	Cytoplasm plasmamembrane and acidic organelles	Acidification of extracellular microenvironment and endo-lysosomal compartment	[21,35]

Na+/H+ ATPase	Cytoplasm plasmamembrane	Alcalinization of cytosol and acidification of extracellular microenvironment	[51]

MCT1 (H+/Lactate symporters)	Cytoplasm plasmamembrane	Elimination of lactate as glucose catabolism product and acidification of extracellular milieu	[52]

Carbonic anhydrase	Cytoplasm plasmamembrane	Regulation of intracellular pH and pH gradients	[53]

H+/K+ ATPase	Gastric parietal cells	Regulation of extratracellular pH	[54,59]

ATP- binding cassette	Cytoplasm and intracellular membranes	Transport and extrusion of chemotherapeutic drugs	[55-58]

## Conclusions

As a rule of thumb it is reasonable to speculate that proton pump inhibitors, being pro-drugs needing acidity to be transformed in the active drug [59], might be more active in the most acidic tumors. Some reports have shown that metastatic tumors are more acidic then primary tumors, but also that solid tumors, either carcinoma or melanomas or sarcomas, are more acidic than systemic tumors (i.e. leukemia). It appears therefore conceivable that proton pump inhibitors might be more active against very malignant, often entirely unresponsive to current therapy, tumors. In support to this hypothesis it has also been shown that metastatic melanoma cells may be grown in acidic condition while cells deriving from primary tumors die when cultured in the same condition, needing longer periods of adaptation to select acid-resistant cells [60]. In fact, acidic condition increases susceptibility of metastatic melanoma cells to proton pump inhibitors [45]. Results of ongoing and future clinical trials hopefully will provide the proof of concept that inhibition of the proton pump may represent a new approach in the war against cancer, by both improving chemotherapy and inducing tumor self-digestion.

In conclusion, proton pump inhibitors might become a crucial addition to the pharmaceutical "armoury" of oncologists in consideration of their low cost, minimal toxicity and high efficacy. Further preclinical and clinical trials are ongoing to provide the clinical proof of concept for the use of proton pump inhibitors in the treatment of malignant cancers.

## Competing interests

The authors declare that they have no competing interests.

## Authors' contributions

All the authors read and approved the final manuscript. EPS and SF equally contributed to this work, GC supervised the other contributors and critically revised the manuscript.
